# The Role of the Gut Microbiota in Individuals with Irritable Bowel Syndrome: A Scoping Review

**DOI:** 10.3390/medicina60111895

**Published:** 2024-11-19

**Authors:** Takahiko Nagamine

**Affiliations:** 1 Department of Psychiatric Internal Medicine, Sunlight Brain Research Center, Hofu 7470066, Japan; tnagamine@outlook.com; Fax: +81-835-25-6610; 2 Department of Psychosomatic Dentistry, Graduate School of Medical and Dental Sciences, Institute of Science Tokyo, Bunkyou 1138510, Japan

**Keywords:** irritable bowel syndrome, brain–gut-microbiota axis, dysbiosis, butyrate-producing bacteria, dopamine, depression

## Abstract

Irritable bowel syndrome (IBS) represents the most prevalent disorder of brain–gut interaction, affecting approximately 10% of the global population. The objective of this study was to examine the mechanisms by which the gut microbiota contributes to the development of IBS. To this end, a review of articles that examined the gut microbiota of IBS patients was conducted. A search was conducted using PubMed and J-STAGE for articles published over the past five years that relate to the gut microbiota in patients with IBS. Individuals diagnosed with IBS display a reduction in alpha diversity and a decline in butyrate-producing bacteria, which collectively indicate a state of dysbiosis within their gut microbiota. Butyrate plays a dual role in the body, acting as a source of nutrition for the intestinal epithelium while also regulating the expression of dopamine transporters and D2 receptors in the central nervous system through epigenetic mechanisms. These characteristics may be linked to dysfunction of the central dopamine D2 pathway and play a role in the formation of various symptoms in IBS.

## 1. Introduction

Irritable bowel syndrome (IBS) is a chronic gastrointestinal disorder that presents with abdominal pain and discomfort, bloating, flatulence, and alterations in bowel habits, including constipation and diarrhea. The global prevalence of IBS is estimated to be approximately 10%, with women exhibiting a prevalence that is 1.5 times higher than that of men. Additionally, the age distribution of IBS cases exhibits three peaks, with the majority occurring in young adults and middle-aged and older adults [1]. The precise mechanism underlying IBS remains unclear. However, it is hypothesized that a complex interplay between various factors, including genetic predisposition, social stress, a history of previous enteritis, and the brain–gut-microbiota axis, may contribute to its pathogenesis. The gut microbiota is a diverse group of microorganisms that inhabit the intestine and play an integral role in a multitude of bodily functions, including digestion, immune response, and mental health [2]. The composition of the gut microbiota modulates the communication between the brain and the gut, which, in turn, affects intestinal motility, intestinal permeability, and visceral hypersensitivity [3]. Alterations in the gut microbiota are postulated to be involved in the onset and persistence of IBS [4]. An analysis of the gut microbiota of IBS patients may elucidate the underlying pathology. The advent of next-generation sequencers has enabled comprehensive examinations of bacterial flora without culturing, thereby facilitating advancements in research on the gut microbiota of IBS patients. In light of the above, the present study offers a comprehensive narrative review of the gut microbiota in individuals with IBS, along with an in-depth examination of the underlying pathogenesis of IBS.

## 2. Materials and Methods

**Figure 1:**
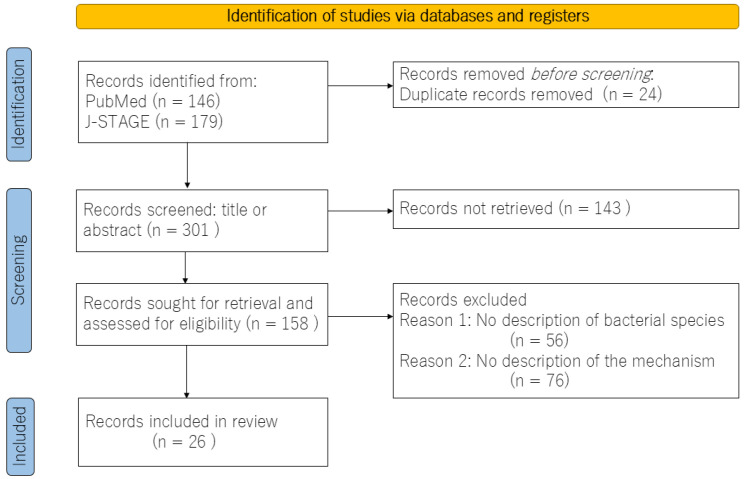
Flow diagram for the review, which included searches of databases and registers.

The objective of this study was to examine the mechanisms by which the gut microbiota contributes to the development of IBS. To this end, a review of articles that examined the gut microbiota of IBS patients was conducted. A comprehensive systematic search of the PubMed and the J-STAGE databases was conducted for articles published in English between January 2019 and March 2024. The search terms employed were “irritable bowel syndrome”, “intestinal flora”, and “mechanism”. The article types included in the search were experimental studies, case reports, comparative studies with healthy subjects, and reviews. The full texts of the retrieved articles were examined and included only if they addressed the mechanisms involved in IBS. The screening and selection process was conducted by the author. Following an initial review of 301 abstracts, it became evident that few studies aligned with the objectives of this study. A flow diagram of the review process, including database and register searches, is shown in Figure 1. In conclusion, a total of 26 studies were included in the review, the majority of which were either meta-analyses or basic experiments. In view of the above, this paper presents a narrative review of recent clinical and experimental findings regarding the mechanisms of IBS from the perspective of the gut microbiota.

## 3. The Gut Microbiota of Patients with IBS

A substantial body of evidence, as demonstrated by numerous meta-analyses, indicates that individuals with IBS often exhibit a distinct composition of gut microbiota compared to healthy controls, accompanied by a reduction in alpha diversity. Alpha diversity is a measure of the diversity of microbial species within a stool sample. It gives an indication of the richness and uniformity of the microbial community. Richness refers to the number of different species present in a sample. The higher the richness, the greater the diversity of microbial species. Evenness refers to the relative abundance of each species. Higher evenness means that species are more evenly distributed, rather than having a few dominant species and many rare species. In general, greater diversity in the gut microbiome is associated with better health. In IBS patients, dysbiosis, a decline in alpha diversity, is typified by diminished levels of beneficial bacteria, such as *Bifidobacterium* and *Lactobacillus*, and elevated levels of potentially deleterious bacteria, including *Bacteroides* and *Enterobacteriaceae* [5]. However, there are notable differences between ethnic groups. For instance, the incidence of propionic acid-producing *Veillonella* is higher among Japanese IBS patients, while the family *Enterobacteriaceae* and genus *Bacteroides* are more prevalent in Northern Europe and the United States IBS patients [6]. Ethnic differences in the gut microbiota of IBS patients are also related to dietary habits and environmental factors. It seems that no specific types of bacteria are exclusively associated with IBS, as the intestinal microbiota is mainly influenced by dietary and host genetic factors [7]. Although there is considerable variation in the specific bacterial species involved, a common feature of patients with IBS is a reduction in butyrate-producing bacteria [8]. Butyrate is a short-chain fatty acid produced by beneficial intestinal bacteria, and it plays a pivotal role in maintaining intestinal health [9]. In light of the high prevalence of gut dysbiosis and the reduction in butyrate-producing bacteria observed in patients diagnosed with IBS, therapeutic interventions have been developed with the specific aim of altering the microbiota. These have included a variety of probiotics and fecal transplants. In prior intervention studies, an increase in butyrate-producing bacteria was associated with an improvement in symptoms [10].

## 4. Examining the Gut Microbiota via the Type of IBS

IBS is classified into four categories: the diarrhea-dominant type, the constipation-dominant type, the mixed type, and unclassified. It has been postulated that discrepancies in monoamine levels, including the levels of serotonin, dopamine, and gamma-aminobutyric acid (GABA), which are implicated in gastrointestinal motility in the digestive tract, may be linked to variations in symptoms observed in individuals with IBS [11]. Nevertheless, there is currently no evidence to support the hypothesis that distinct types of monoamine-producing bacteria are associated with disparate forms of IBS [12]. *Methanobrevibacter smithii* has been identified as a potential contributing factor in the development of the constipation-predominant type, given its ability to produce methane and prolong intestinal transit time [13]. However, this bacterial species is not prevalent in many IBS patients, and differences in IBS types are largely influenced by sex. The constipation-predominant type is more prevalent in women, whereas the diarrhea-predominant type is more common in men [14]. The regulation of colonic motility via the central nervous system is mediated by the descending pain inhibitory pathway, which exhibits sex-specific differences in its neural circuitry. Noxious stimuli in the colon of anesthetized male rats have been observed to activate monoamine neurons in the descending pain inhibitory pathway, which extends from the brainstem to the lumbosacral spinal cord. This appears to enhance colonic motility. The descending pain inhibitory pathway in male rats releases serotonin and dopamine into the lumbosacral spinal cord, thereby enhancing colonic motility. In contrast, noxious stimuli in the colon have minimal impact on colonic motility in female rats. In female rats, the descending pain inhibitory pathway releases serotonin and GABA into the lumbosacral spinal cord. GABA masks the enhancement of colonic motility by serotonin, as evidenced by prior research [15]. The type of IBS may be influenced by sex hormones that affect the expression of monoamines in the descending pain inhibitory pathway [16].

IBS not only causes bowel problems but also causes unexplained abdominal pain, and sex hormones may be involved in the formation of this pain. Furthermore, the observation that IBS manifests in two distinct age groups, namely in those experiencing adolescence and menopause, may be associated with the fluctuations in estrogen levels. The hypothesis that painful disorders occur in two phases, as estrogen levels increase and then decrease, is known as the “two-hit theory by estrogen” [17]. The expression of the transient receptor potential vanilloid 1 (TRPV1), a nociceptive receptor, is increased by estrogenic effects and is associated with increased pain sensitivity. As a preliminary impact of estrogen, elevated estrogen levels during puberty enhance TRPV1 expression, thereby increasing pain sensitivity. Conversely, estrogen has been demonstrated to alleviate pain by downregulating nerve growth factor (NGF), which has the potential to translocate TRPV1 receptors to the cell surface membrane [18]. As a second impact of estrogen, a decrease in estrogen during menopause results in an increase in NGF, an increase in TRPV1 receptors at the cell surface, and an increase in pain sensitivity in individuals at risk for developing pain.

It has been postulated that constipation-type IBS may be related to female sex hormones. Although the specific type of IBS is significantly influenced by sex hormones, these hormones also impact the composition of the intestinal microbiota, suggesting a potential indirect role for the gut microbiota in the development of IBS. The gut microbiota can exhibit sexual dimorphism, indicating the potential influence of sex hormones, and it has also been demonstrated to undergo alterations with age [19]. Constipation-predominant IBS is frequently observed in menopausal women, a condition that is markedly influenced by the decline in female hormones during menopause. Moreover, since female hormones have the effect of maintaining the diversity of the intestinal microbiota, the decrease in female hormones during menopause reduces alpha diversity [16], which, in turn, leads to dysbiosis and affects the onset of IBS. It is regrettable that there is currently no evidence to suggest whether diarrhea-predominant IBS is associated with male-type gut microbiota.

## 5. The Relationship Between the Gut Microbiota and Nociplastic Pain

IBS is a visceral pain disorder that is associated with nociplastic pain [20]. Nociplastic pain is a form of chronic pain that arises from altered pain processing in the central nervous system, rather than from direct damage to tissues or nerves [21]. The composition of the gut microbiota may be associated with the development of nociplastic pain conditions including chronic orofacial pain, complex regional pain syndrome, fibromyalgia, and IBS [22]. However, no specific bacterial species is consistently associated with all forms of nociplastic pain. The composition of the gut microbiota also varies between individuals with chronic pain diseases. However, nociplastic pain does exhibit some commonalities in the intestinal bacterial profile, including a low prevalence of butyrate-producing bacteria and a leaky gut as a underlying mechanism [23]. A meta-analysis of the gut microbiota in patients with chronic pain disorders, including a significant number of cases of nociplastic pain, revealed that there were no disease-specific alterations in the gut microbiota, but rather a reduction in alpha diversity. While numerous beneficial bacteria, including the butyrate-producing *Lachnospiraceae* family, which may also possess probiotic properties, exhibited a decline, the opportunistic *Eggerthella* spp. demonstrated an increase in [24]. *Eggerthella* spp. encompass numerous bacterial species that have the potential to alter enzyme activity. For instance, they may enhance dopamine metabolism, leading to dopamine depletion [25], or they may enhance estrogen function as part of the estrobolome [26]. Although the precise mechanism of nociplastic pain remains unclear, a meta-analysis of the gut microbiota in patients with chronic pain disorders of unknown etiology suggests that a reduction in dopamine and an increase in estrogen action may influence the nervous system’s involvement in central pain perception [24,25].

## 6. The Relationship Between the Gut Microbiota and Dopamine

A substantial body of evidence has emerged suggesting a potential role for central dopamine pathways in the pathogenesis of IBS. In a rat IBS model in which colonic hyperpermeability was induced using proinflammatory cytokines, visceral allodynia and colonic hyperpermeability were improved by the anti-inflammatory drug losartan. However, this effect was abolished by sulpiride, a dopamine D2 receptor antagonist, suggesting that dopamine D2 pathways are involved in both intestinal permeability and visceral pain [27]. The dopamine D2 pathway is implicated in both leaky gut and visceral pain in the pathophysiology of IBS.

The production of neurotransmitters by gut bacteria has been proposed as a mechanism through which the gut microbiota can alter central nervous system function. Additionally, alterations in the composition of gut bacteria and the central dopamine pathways have been attributed to the production of monoamines by the gut microbiota, which are analogous to those found in the nervous system, including glutamate, serotonin, norepinephrine, and GABA [28]. However, the existence of the blood–brain barrier precludes the possibility that the gut microbiota directly reduces the function of the central dopaminergic nervous system by reducing dopamine biosynthesis. Therefore, rather than regulating the ability of the gut microbiota to produce monoamines, it is necessary to consider that a signal emitted by the gut microbiota changes the function of the dopaminergic nervous system.

Individuals diagnosed with IBS have been observed to exhibit diminished levels of butyrate-producing bacteria. Butyrate is the preferred energy source for the cells that line the colon, known as colonocytes. It is easily absorbed and utilized by these cells. Butyrate regulates bowel motility and helps reduce symptoms of constipation and diarrhea. Butyrate helps maintain the integrity of the tight junctions between the colonocytes. This prevents harmful substances from entering the bloodstream, reduces inflammation, and improves overall gut health. In addition, butyrate can influence the composition of the gut microbiota by promoting the growth of beneficial bacteria and inhibiting the growth of harmful bacteria [29]. Butyrate, in turn, has been demonstrated to regulate the function of the central dopamine nervous system through epigenetic effects. Butyrate has been demonstrated to potently inhibit heterochromatin formation by inhibiting histone deacetylase (HDAC), thereby relaxing chromatin and increasing its susceptibility to gene expression. In a mouse model of Parkinson’s disease, it has been demonstrated that histone deacetylase (HDAC) inhibition by butyrate increases striatal dopamine levels and improves motor function [30]. Moreover, the increase in intestinal butyrate resulting from the action of butyrate-producing bacteria has been observed to lead to an increase in the production of the gut hormone glucagon-like peptide-1 (GLP-1), which, in turn, has been shown to result in an increase in the expression of the GLP-1 receptor in the gut. Increased GLP-1 signaling by butyrate-producing bacteria has been demonstrated to confer neuroprotective benefits and improve neurobehavioral deficits in animal models exhibiting reduced dopamine neurotransmission [31]. In the striatum, the expression of dopamine transporter (DAT) and D1/D2 receptors is altered by gut bacteria. Among the several bacteria that have been found to be decreased in the gut microbiota in IBS, *Prevotella*, *Bacteroides*, *Lactobacillus*, *Bifidobacterium*, *Clostridium*, *Enterococcus*, and *Ruminococcus* are known to increase DAT expression and D1/D2 receptor expression and improve dopamine neurotransmission [32]. The decrease in these beneficial flora in IBS may cause a decrease in the function of the dopamine nervous system.

## 7. Symptoms Caused by the Gut Microbiota or Genetics

It is estimated that approximately one-third of individuals with IBS also experience symptoms of anxiety or depression [33]. These conditions exhibit a bidirectional relationship, with each influencing the pathology of the other. A meta-analysis of the gut microbiota of patients with depression and anxiety revealed that they exhibited elevated levels of inflammation-inducing bacteria, including *Enterobacteriaceae* and *Desulfurovibrio*, and diminished levels of *Faecalibacterium*, a bacterium that produces short-chain fatty acids, such as butyrate [34]. Anxiety, depression, and IRB share a common gut microbiota pattern of gut dysbiosis and reduced butyrate-producing bacteria. Although the bacterial species are not identical, the characteristics of the gut microbiota are consistent. Additionally, it has been proposed that the high prevalence of psychiatric comorbidities in IRB patients may be influenced by the gut–brain-microbiota axis, which refers to the interaction between the intestinal flora and the central nervous system.

However, a genome-wide association study identified six genetic susceptibility loci for IBS—NCAM1 (neural cell adhesion molecule 1), CADM2 (cell adhesion molecule 2, PHF2/FAM120A (PHD finger protein 2/family with sequence similarity 120 member A), DOCK9 (dedicator of cytokinesis 9), CKAP2/TPTE2P3 (cytoskeleton-associated protein 2/transmembrane protein with EGF-like and two follistatin-like domains 2 pseudogene 3), and BAG6 (BCL2-associated athanogene 6)—four of which are also associated with mood and anxiety disorders [35]. NCAM1 and CADM2 have been demonstrated to influence the formation and functionality of neural circuits between the brain and the gut, which may contribute to the development of intestinal hypersensitivity and aberrant motility. It is postulated that CADM2 may affect the junctions between cells in the intestinal mucosa, thereby increasing intestinal permeability and causing inflammation. PHF2/FAM120A may induce alterations in intestinal motility and sensory hypersensitivity by modulating the expression of genes involved in intestinal function. DOCK9 and CKAP2/TPTE2P3 have been demonstrated to enhance intestinal inflammatory responses. BAG6 is a gene that encodes a molecular chaperone and is involved in regulating cellular quality control. It may also influence the stress response of enterocytes. The genetic predisposition to impaired cell–cell adhesion and the diminished functionality of molecular chaperones associated with proinflammatory processes and their repair may be a key factor in the pathogenesis of IBS. Against this genetic background, the gut microbiota may contribute to the formation of IBS symptoms by increasing intestinal permeability and reducing butyrate that protects the mucosa. It is also a possibility that IBS and anxiety/depression coexist through a common genetic locus. The host genetic background influences the development of IBS and depression symptoms, but it may also affect gut microbiota development, including dysbiosis and a reduction in butyrate-producing bacteria. However, the gut microbiota may be more influenced by diet and living environment than by the host’s genes. Therefore, some symptoms may be influenced more by genes, while others may be influenced more by the gut microbiota.

**Figure 2:**
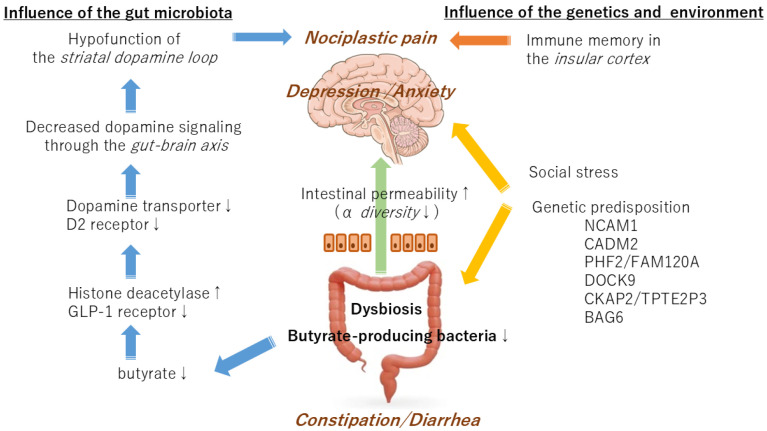
A schematic diagram of the interaction of the gut microbiota, genetic factors, and environmental factors in the development of irritable bowel syndrome.

It should be noted that autism is a psychiatric disorder that is associated with gut dysfunction and is related to the brain–gut-microbiota axis. Experiments employing contactin-associated protein-like 2 knockout mice, a model for autism, have demonstrated that the hyperactivity observed in autism is genetically determined and that the lack of sociability is caused by intestinal bacteria [36]. It has been demonstrated that mice models of autism exhibit symptoms resulting from genetic and microbial factors. Thus, some symptoms of IRB may be regulated by gut bacteria via the brain–gut axis, whereas other symptoms are more likely to be due to genetic factors. Although the coexistence of gastrointestinal inflammation and depression in IBS may be largely genetically controlled, the pain pathways in the brain that determine how pain is felt may be a symptom of the gut microbiota (Figure 2).

It has been established that an important risk factor for IBS that is not attributable to genetic factors is the occurrence of infectious enteritis, such as dysentery [37]. The precise mechanism by which a history of enteritis, but not current enteritis, leads to IBS symptoms remains unclear. Using activity-dependent cell labeling techniques in mice, it has been confirmed that memory of intestinal inflammation is stored in the insular cortex. After the intestinal inflammation subsided, stimulation of the insular cortex cells using the chemogenetic reactivation method resulted in a relapse of the intestinal inflammation, even in the absence of the inflammation-causing substance [38]. The insular cortex, in conjunction with the cingulate gyrus, constitutes a salience network, a large-scale brain network that is activated by anxiety and pain. This network is regulated by the dopamine nervous system in the medial prefrontal cortex [39]. Changes in the function of dopamine neurons in the mesolimbic system may regenerate the pain memory of enteritis stored in the insular cortex while simultaneously inducing anxiety neural circuits. The interactions of the gut microbiota with genetic and environmental factors may result in the manifestation of various symptoms of IBS through alterations in the function of the dopaminergic nervous system.

## 8. Limitations

It should be noted that this study is subject to a number of limitations. Firstly, the methodologies employed to comprehensively examine the gut microbiota in IBS patients in the reviewed papers lack standardization. Consequently, the veracity of the analytical outcomes is contingent upon the caliber of the reference database employed. The possibility exists that new bacterial species not registered in the database may not be detected. Moreover, few studies considered the impact of dietary and lifestyle habits on the gut microbiota. There was minimal discussion of the relationship between the detected gut bacteria and the mechanism of IBS. Additionally, the majority of reports on mechanisms by bacterial species were derived from animal experiments. Notwithstanding these limitations, this report proposes that the gut microbiota of IBS patients may be a contributing factor in the pathogenesis of nociplastic pain by modulating the function of the dopamine nervous system in conjunction with host genes, which may serve as a guiding principle for future research endeavors.

## 9. Conclusions and Future Prospects

IBS is a disease characterized by nociplastic pain resulting from alterations in the processing of pain signals in the brain, without any organic damage to the gastrointestinal tract. The pathogenesis of IBS is shaped by the interaction of host genes, stress, and the gut microbiota, which is characterized by dysbiosis and decreased butyrate-producing bacteria. This feature may be responsible for the reduced function of dopaminergic neurotransmission in the central nervous system.

The administration of butyrate-producing bacteria, such as *Clostridium butyricum*, has been demonstrated to improve intestinal immune responses and ameliorate symptoms in animal models of IBS [40]. Indeed, complex probiotics (BioThree) comprising *Clostridium butyricum* have been demonstrated to enhance immune equilibrium by influencing peripheral blood mononuclear cells and dendritic cells [41]. Further research is required to ascertain the efficacy of these probiotics in patients with IBS, with the function of the central dopamine nervous system included as an evaluation index. A prospective randomized trial would be beneficial for determining whether administration of a probiotic containing butyrate-producing bacteria can improve the balance of the dopaminergic nervous system in the brain and effectively alleviate IBS symptoms.

## Data Availability

The data presented in this study are only available upon request from the corresponding author due to the safeguarding of analytical methods.
